# The impact of BMI on disease activity and growth outcomes in juvenile idiopathic arthritis

**DOI:** 10.1007/s00431-025-06084-x

**Published:** 2025-03-19

**Authors:** Çisem Yıldız, Batuhan Küçükali, Merve Kutlar, Nuran Belder, Nihal Karaçayır, Büşra Acun, Pelin Esmeray Şenol, Emine Nur Sunar Yayla, Deniz Gezgin Yıldırım, Sevcan A. Bakkaloğlu

**Affiliations:** 1 Department of Pediatric Rheumatology, Gazi University Faculty of Medicine, Besevler, Ankara, 06560 Turkey; 2 Department of Pediatric Rheumatology, Mersin City Hospital, Mersin, Turkey; 3 Department of Pediatric Rheumatology, Etlik City Hospital, Ankara, Turkey

**Keywords:** Juvenile idiopathic arthritis, Body mass index, Growth, Disease activity

## Abstract

Juvenile idiopathic arthritis (JIA) presents with diverse phenotypes and can lead to significant morbidity. Obesity, affecting 5 to 23% of JIA patients, may exacerbate disease activity and complicate management. This study evaluates the impact of body mass index (BMI) on disease activity and the influence of joint involvement on BMI in JIA patients. Between January 2012 and June 2024, 225 JIA patients were reviewed, with 173 ultimately included based on specific inclusion and exclusion criteria. Treatments followed the American College of Rheumatology (ACR) recommendations. Data on demographics, BMI, JADAS-27 scores, joint involvement, and laboratory parameters were analyzed, with statistical significance defined as *p* < 0.05. Among 173 JIA patients, significant increases in weight and height SDS were observed (*p* < 0.05). Obese patients at baseline had higher JADAS-27 scores at 6 months (*p* < 0.05). A positive correlation was found between initial JADAS-27 and final BMI SDS (*r* = 0.170, *p* < 0.05). Patients with hip involvement had lower BMI at diagnosis and at the first-year follow-up (*p* < 0.0001, *p* = 0.049), while knee involvement was linked to lower height SDS at the second year (*p* = 0.041).

*Conclusion*: Our study revealed that baseline obesity is a significant risk factor for poorer disease control at the 6-month follow-up in JIA patients. We also observed that effective treatment led to improvements in growth, particularly in patients with hip involvement, who initially had lower BMI values. These findings underscore the importance of monitoring BMI in JIA patients to optimize disease management and long-term outcomes.

**Table:** 

**What is Known:** • *Overweight and obesity are prevalent in children with JIA, with reported rates ranging from 5 to 23% in various studies.* • *The relationship between BMI and disease activity in JIA remains controversial, with previous studies reporting conflicting results on whether BMI impacts disease remission or activity.*	
**What is New:** • *Baseline obesity in juvenile idiopathic arthritis (JIA) patients is associated with worse disease activity during follow-up, particularly evident at the 6-month visit.* • *Patients with hip involvement had significantly lower BMI values, indicating a potential correlation with more severe disease.* •* Improved linear growth was observed in JIA patients during follow-up, indicating that effective treatment positively impacts growth despite the chronic nature of the disease.*

**Supplementary Information:**

The online version contains supplementary material available at 10.1007/s00431-025-06084-x.

## Introduction

Juvenile idiopathic arthritis (JIA) is the most common chronic rheumatic disease in childhood and can lead to significant short- and long-term morbidity [1–6]. It encompasses a heterogeneous group of diseases with varying phenotypes, clinical course, and prognosis [4, 6]. Prior to the introduction of new and effective treatments, malnutrition symptoms such as stunted growth, low weight, and muscle wasting were common among JIA patients [7–9]. In recent years, novel therapeutic options have significantly improved long-term outcomes [1, 4]. Chronic inflammation in children with JIA can lead to growth delay and poor weight gain, while corticosteroids used to control inflammation may also cause growth delay and excessive weight gain [10].

Overweight and obesity are prevalent medical issues affecting children and adolescents worldwide [11–13]. The World Health Organization (WHO) defines overweight and obesity as abnormal or excessive fat accumulation that may impair health, and their rates continue to rise among both adults and children [13, 14]. Globally, obesity rates have quadrupled from 2 to 8% between 1990 and 2022 [14]. According to the latest data from the Centers for Disease Control and Prevention (CDC), obesity is considered a serious public health issue requiring urgent intervention and strategies [13]. Children with physical disabilities are at a higher risk of obesity, and the prevalence of overweight and obesity in patients with JIA ranges from 5 to 23% [13, 15].

There is evidence that obesity increases the risk of numerous cardiometabolic, pulmonary, and psychosocial complications for both adults and children [11]. Body mass index (BMI) is the most commonly used method to define obesity and overweight; however, it does not account for body composition and the distribution of fat tissue [16]. Additionally, elevated BMI is associated with changes in the biomechanical and inflammatory environments of joints [11, 17]. Previous studies have shown that obesity in adults is linked to a decreased likelihood of achieving remission and a reduction in quality of life in rheumatoid arthritis (RA) [18]. Increased body weight has also been found to be associated with worsening disease activity in RA, ankylosing spondylitis (AS), and psoriatic arthritis (PsA) [18–20]. A cross-sectional study indicated that, in addition to obesity, being underweight is also associated with increased RA disease activity [21]. However, the relationship between BMI and disease activity in JIA has not been sufficiently investigated [4]. This study aims to evaluate the impact of BMI levels on disease activity in JIA patients and the effect of joint involvement on BMI.

## Methods

### Patient selection

Between January 2012 and June 2024, the medical records of 225 patients diagnosed and classified with JIA according to International League of Associations for Rheumatology (ILAR) criteria [22] at the Gazi University Faculty of Medicine, Pediatric Rheumatology Department, were retrospectively reviewed. Approval for the study was obtained from the Gazi University Ethics Committee. Patients were excluded if they had incomplete data (e.g., missing height, weight, and BMI measurements at baseline or follow-up visits), missed three or more consecutive scheduled follow-ups, had a follow-up period of less than 6 months, or had underlying diseases. Consequently, a total of 173 patients were included in this retrospective observational study.

Treatment for JIA was administered in accordance with the American College of Rheumatology recommendations available at the time of each patient’s treatment initiation or modification [23–25]. At our center, non-steroidal anti-inflammatory drugs (NSAIDs) and intra-articular steroids (IACIs) are the first-line treatments for patients with oligoarthritis. Conventional and biologic disease-modifying anti-rheumatic drugs (cDMARDs, bDMARDs) are utilized for children with systemic or polyarticular JIA, or for those with oligoarthritis who do not respond to NSAIDs or IACIs.

### Data collection

All data was collected using a standardized form until June 2024. Basic demographic data, including age at symptom onset, age at diagnosis, gender, JIA category, and medical treatments received, were obtained from patient’s electronic medical records. The confirmation of the patient’s diagnosis was considered the baseline, and subsequent data were retrospectively collected from medical records at 6th month, 1st year, 2nd year, and the last follow-up visit. BMI SDS values and JADAS-27 at diagnosis and during follow-up (baseline, 1st year, 2nd year, and last visit), affected joints, and laboratory parameters (erythrocyte sedimentation rate [ESR], c-reactive protein [CRP]) were recorded. Disease activity was calculated using the JADAS-27 [26], which includes combining scores from the patient visual analog scale (VAS), the physician VAS, the active joint count, and the levels of acute phase reactants (ESR, CRP), each rated on a scale of 0 to 10. Weight (in kilograms) and height (in centimeters) were measured by appropriately trained medical personnel at each clinical visit, and BMI was calculated in kg/m^2^ for each visit. Weight, height, and BMI values were transformed into age- and sex-specific scores (SD scores) according to the 2000 data from the US CDC [27]. BMI measurements were also categorized as normal (5th–85th percentile), overweight (85th–95th percentile), or obese (≥ 95th percentile) according to established age- and sex-specific cut-off points. Medication exposures were assessed as “any use” during the follow-up period, meaning that the data refers to whether a patient had used a specific medication at any point during the follow-up. The study adheres to the principles outlined in the Declaration of Helsinki, and approval for the protocol was obtained from the institutional ethics committee.

### Statistical analysis

All statistical modeling was conducted using IBM SPSS software version 23. Variables were presented as mean ± standard deviation (SD) or median (interquartile range) according to the distribution of the variable; for categorical variables, number and percentage values were presented. Differences in baseline characteristics among groups were compared using the chi-square test for categorical variables. Spearman’s rho test was used to assess linear correlation between non-parametric variables. Differences in continuous variables were analyzed using the independent-sample *t*-test for normally distributed variables, and the Mann–Whitney test when normality assumptions were not met. The paired *t*-test and Wilcoxon test was employed to compare the means between two dependent groups within the dataset. To assess the effect of multiple independent variables on one or more dependent variables, a multivariate analysis was conducted using multiple regression analysis and MANOVA. The confidence intervals were calculated at the 95% confidence level, and differences at *p* < 0.05 were considered statistically significant.

### Ethical considerations

All data were kept anonymous and encrypted. This study was conducted in accordance with the Helsinki Declaration. Ethics Committee approval was obtained from Gazi University (Decision number: E-77082166–604.01–1052477 / 2024–1482 dated 24.09.2024).

## Results

**Fig. 1:**
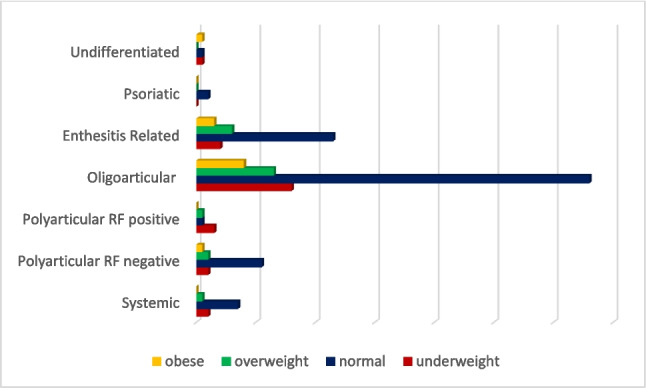
Distribution of BMI categories at the time of diagnosis according to the ILAR classification

A total of 173 patients with a median age at diagnosis of 9.20 years (IQR = 5.5–9.37) were included in the study. Female-to-male ratio was 1.27 (56% (*n* = 97) female, 44% (*n* = 76) male (*p* > 0.05). The total follow-up duration was 60.6 ± 39.9 months. The median BMI at diagnosis was 17 kg/m^2^ (IQR = 14.85–19.87) with 15% (*n* = 26) classified as underweight, 64% (*n* = 111) as normal weight, 13% (*n* = 23) as overweight, and 8% (*n* = 13) as obese (Table 1). The distribution of BMIs according to ILAR JIA categories is shown in Fig. 1. Strikingly, the majority of the patients in the polyarticular JIA group were classified as underweight, whereas in the other JIA categories, except for the undifferentiated group, most patients had a normal BMI.

**Table 1:** Patient characteristics

Gender	Female	56.1% (*n* = 97)
	Male	43.9% (*n* = 76)
Age at diagnosis (median [IQR]) (years)		9.20 (5.5–9.37)
The age at onset of complaints (median [IQR]) (years)		8.89 (5.15–12.42)
The duration of diagnostic delay (median [IQR]) (months)		2 (0.5–7.0)
ILAR subcategories		
Oligoarticular JIA		58.4% (*n* = 101)
Enthesitis-related arthritis		20.8% (*n* = 36)
Polyarticular (RF-negative) JIA		9.2% (*n* = 16)
Polyarticular (RF-positive) JIA		2.9% (*n* = 5)
Systemic JIA		5.8% (*n* = 10)
Psoriatic arthritis		1.2% (*n* = 2)
Undifferentiated		1.7% (*n* = 3)

*IQR* interquartile range

**Fig. 2:**
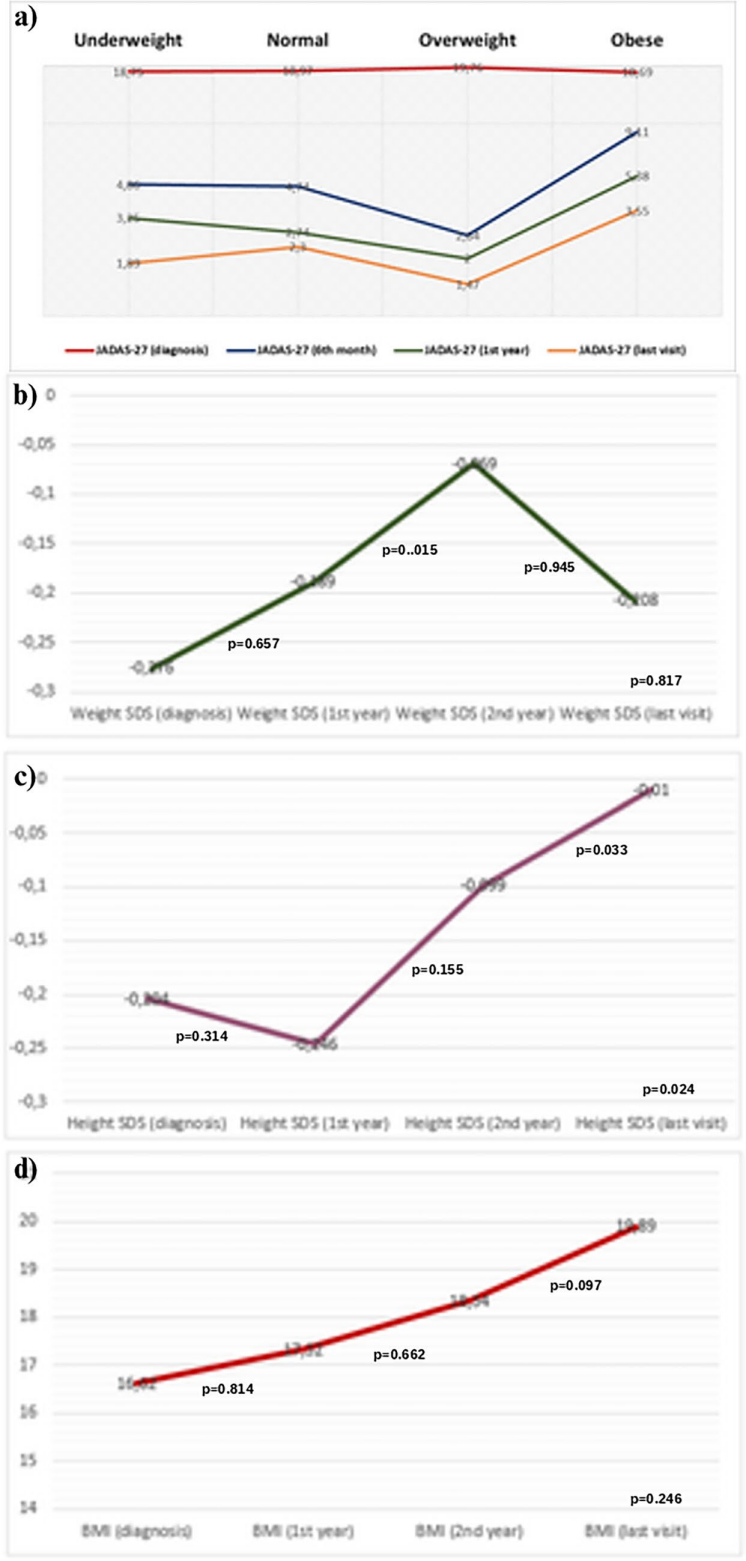
**a** The change in patients’ JADAS-27 scores over time according to initial BMI. **b** Longitudinal variations in weight among patients over time. **c** Longitudinal variations in height among patients over time. **d** Longitudinal variations in BMI among patients over time

Figure 2 illustrates the changes in weight, height, and BMI of the patients over time. We observed a significant increase in the weight SDS values between the first and second year (*p* < 0.05) (Fig. 2b). The increase in the median height SDS values was observed both between the second year and final follow-up, from baseline to the final follow-up (*p* < 0.05, *p* < 0.05 respectively) (Fig. 2c). Figure 2a illustrates that patients who were overweight at baseline had lower JADAS-27 scores at follow-up visits (6th month, 1st year, last visit) compared to patients who were underweight, of normal weight, or obese. Conversely, it was observed that patients who were obese at baseline had higher JADAS-27 scores during follow-up (6th month, 1st year, last visit). Multivariate analysis indicated that, at the 6-month follow-up, patients who were initially obese demonstrated significantly higher JADAS-27 scores compared to those who were initially normal weight or overweight ((Mean difference = 4.3733, *p* = 0.035, 95% CI [0.1873, 8.5592]), (Mean difference = 6.4683, *p* = 0.007, 95% CI [1.2530, 11.6836]), respectively) (Table 2, Fig. 2a). When evaluating the relationship between BMI and JADAS-27 at diagnosis, during the first and the second years of follow-up, and at the last control visit, a statistically significant positive correlation was found between higher initial JADAS-27 scores and the BMI SDS values at the final control visit (*r* = 0.170, *p* < 0.05) (Table 3). In the correlation analysis conducted specifically for patients with oligoarticular JIA, a low positive correlation was found between higher BMI SDS at diagnosis and JADAS-27 at diagnosis (*r* = 0.211, *p* < 0.05) (Table 4).

**Table 2:** The median values and multivariate analysis of JADAS-27 scores according to BMI categories at the time of diagnosis

	Underweight*	Normal*	Overweight*	Obese*	*p*-value^§^
JADAS-27 (diagnosis) (median [IQR])	19 (14.5–22.5)	18 (17–21)	19 (18–20)	19 (17–23)	NS
JADAS-27 (6th month) (median [IQR])	4 (0.12–9)^a^	**2 (0–9)**^**b**^	**1 (0–2.5)**^**c**^	**9 (1.25–14.5)**^**d**^	^a–b, a–c, a–d, b–c^ NS ^**b–d**^**0.035** ^**c–d**^**0.007**
JADAS-27 (1st year) (median [IQR])	0 (0–5)	1 (0–5)	0 (0–3)	4 (1–7.5)	NS
JADAS-27 (last visit) (median [IQR])	0 (0–1.87)	0 (0–2)	0 (0–2.5)	1 (0–6)	NS

*JADAS* Juvenile Arthritis Disease Activity Score, *IQR* interquartile range, *NS* not significant

^*^At the time of diagnosis, ^§^post hoc analysis, multivariate analysis-significance level *p* < 0.05

**Table 3:** Correlation between disease activity and JADAS-27

	Spearman’s correlation	BMI SDS (at diagnosis)	BMI SDS (1st year)	BMI SDS (2nd year)	BMI SDS (last visit)
JADAS-27 (diagnosis)	r	0.72	0.060	0.129	0.170*
	p	0.345	0.479	0.163	0.028*
	n	173	143	119	167
JADAS-27 (6th month)	r	0.26	0.054	−0.031	− 0.098
	p	0.745	0.528	0.744	0.214
	n	165	140	116	161
JADAS-27 (1st year)	r	0.005	0.034	0.105	−0.022
	p	0.947	0.693	0.262	0.794
	n	151	137	117	147
JADAS-27 (last visit)	r	0.078	0.029	0.035	0.016
	p	0.312	0.733	0.705	0.838
	n	171	142	119	166

*Significance level *p* < 0.05

**Table 4:** Correlation analysis of BMI SDS and JADAS-27 in patients with oligoarticular JIA

		JADAS-27 (diagnosis)	JADAS-27 (6th month)	JADAS-27 (1st year)	JADAS-27 (last visit)
BMI (SDS) (diagnosis)	Correlation coefficient*	**0.211**	0.50	− 0.006	0.076
	*p*-value**	**0.035**	0.629	0.955	0.455
	*N*	100	95	89	100
BMI (SDS) (1st year)	Correlation coefficient*	0.103	0.157	0.057	0.080
	*p*-value**	0.346	0.159	0.613	0.468
	*N*	85	82	82	85
BMI (SDS) (2nd year)	Correlation coefficient*	0.196	0.125	0.101	0.065
	*p*-value**	0.102	0.307	0.411	0.588
	*N*	71	69	69	71
BMI (SDS) (last visit)	Correlation coefficient*	0.118	− 0.017	− 0.028	0.054
	*p*-value**	0.251	0.870	0.795	0.602
	*N*	97	93	87	97

^*^Spearman’s rho

^**^Significance level *p* < 0.05

Upon assessment of the SDS values of body weight, height, and BMI measurements throughout the patients’ follow-up (at diagnosis, the first year of follow-up, the second year of follow-up, and the final evaluation), it was observed that patients with hip involvement had significantly lower BMI values at the time of diagnosis and during the first-year control compared to those without hip involvement (*p* < 0.0001, *p* = 0.049, respectively) (Supplementary Table 1). In patients with knee involvement, height SDS values at the second year of follow-up were significantly lower compared to those without knee involvement (*p* = 0.041); however, these patients were still within normal height ranges for their age and did not warrant further investigation for short stature. In patients with ankle involvement, body weight at the final control visit was higher than in those without ankle involvement, though these patients did not meet the criteria for obesity.

When evaluating changes in BMI SDS (decreased, same, increased) according to the treatments received by the patients (NSAIDs, cDMARDs anti-TNF, anti-IL-6, steroids), no statistically significant differences were observed between the groups (Supplementary Table 2). In addition, we did not find any significant differences between initial acute phase reactants (ESR, CRP) and BMI values during the follow-up of patients (at diagnosis, the first year of follow-up, the second year of follow-up, and the final control) (Supplementary Table 2).

## Discussion

In our study, we examined the effects of weight, height, and BMI parameters in 173 JIA patients over a period of approximately 12 years, regularly monitored for disease activity and affected joint regions. We found that patients who were obese at the outset had worse JADAS-27 responses at the 6th month visit compared to those who were normal weight or underweight. Additionally, we observed improvement in weight and height SDS changes with treatment. Our findings demonstrated a positive correlation between baseline BMI SDS and disease activity and with patients exhibiting hip involvement displaying a significantly higher BMI SDS at the 1-year follow-up compared to those without.

Examining the BMI levels at the time of diagnosis revealed that 15% of the patients were underweight, 64% were within the normal range, and 21% were overweight or obese. Various rates have been reported in other studies, such as Makay et al. reported that 27.8% of the patients with only enthesitis-related arthritis (ERA) subtype were above normal weight [11]. Another study assessing BMI and disease activity in 40 JIA patients found that 30% of JIA patients were overweight/obese [7]. Additionally, a multicenter study evaluating 275 patients reported that 25.8% were overweight/obese [4], Sherman et al. reported 21.5% [2], and Pelajo et al. reported 30% [13]. Taken together, the prevalence of overweight/obesity in our patient group was slightly lower than previously reported rates.

The relationship between BMI measurements and disease activity in JIA has been evaluated in several studies. Grönlund et al. found no significant differences in anthropometric measurements and nutrient energy intake between children with active and inactive disease [7]. Makay et al. reported that an increase in BMI was associated with a failure to achieve inactive disease status in ERA patients [11]. Conversely, another study evaluating 275 patients found that underweight patients had significantly higher JADAS-27 scores compared to normal weight, overweight, and obese patients [4]. In 2012, Pelajo et al. found no relationship between BMI and JADAS-27 [13]. Evaluation of 110 JIA patients revealed no significant differences between BMI and ESR, CRP, or the total number of active joints; however, lower remission rates were observed in overweight and obese patients, although this relationship was not statistically significant [16]. Gicchino et al. reported that extremely obese and underweight patients had higher JADAS-10, ESR, CRP, and ferritin levels compared to normal weight, overweight, and obese patients [5]. In our study, a positive correlation was found between the initial JADAS-27 and BMI values at the final follow-up. Additionally, patients who were obese at baseline were found to have higher JADAS-27 values at the final follow-up compared to those with normal or overweight, and the difference was statistically significant. Interestingly, the JADAS-27 scores of patients who were overweight at baseline exhibit a U-shaped pattern at the 6-month, 1-year, and final follow-up visits. This may be explained by the “obesity paradox”: despite the negative effects of excessive weight and obesity, recent observational studies have suggested that some individuals with excess weight may have a survival advantage over those of normal weight and underweight, particularly in patients with osteoporosis, cardiovascular disease, chronic kidney disease, and type 2 diabetes mellitus [28–30]. Various mechanisms have been proposed to explain the obesity paradox [28–31]; however, when considering patients with JIA, there is no clear evidence. Whether a modest body reserve at the onset of chronic disease is associated with better outcomes during disease progression remains unclear and can only be elucidated through further investigation. Due to the heterogeneity of the group, the evaluation in the largest subgroup, oligoarticular JIA patients, revealed that patients with higher BMI at diagnosis had higher JADAS-27 (*r* = 0.211, *p* < 0.05). Indeed, we did not find any association between acute phase reactants including CRP and ESR and BMI values.

When evaluated according to the site of involvement, patients with hip involvement had lower BMI values compared to those without hip involvement. This can be explained by two factors: first, hip involvement is generally associated with more severe disease [32–34]. The term “rheumatoid cachexia” has been used to describe the body composition of adult RA patients and is associated with TNF-α, a cytokine known as “cachexin” [1, 35]. TNF-α has been linked to increased resting energy expenditure and anorexia [36]. Therefore, the use of TNF inhibitors (TNFi) has been investigated for its potential impact on a patient’s body mass and composition; however, Shafferman et al. did not demonstrate significant weight gain associated with TNFi treatment [1]. Similarly, in our study, no increase in BMI values was observed in patients receiving TNFi (Supplementary Table 2). For patients with knee involvement, the average height SDS values at the second-year follow-up were lower than those without knee involvement, though still within normal limits (Supplementary Table 1). In a study evaluating anthropometric measurements, biochemical and clinical assessments, and nutrient energy intake in 40 JIA patients, JIA patients were found to have higher weight and adiposity based on body composition compared to matched controls; however, no difference was observed in height SDS values, and there was no association with disease activity [7]. A study evaluating 1497 patients across 16 different centers revealed that the growth of JIA patients was similar to that of healthy children [10]. In the literature, no significant difference in height velocity has been found for JIA patients, indicating that more information is needed to assess this relationship comprehensively. Our data demonstrated that patients, particularly during longitudinal follow-up, exhibited improved height velocity and this indicates that the effective treatment positively impacts linear growth. An analysis of BMI z-scores in 204 JIA patients, both those receiving TNFi and those not, revealed a small but statistically significant increase over an average of 2.8 years; however, this increase did not differ significantly from that observed in the comparison cohort [1]. Similarly, a single-center cross-sectional study involving 154 JIA patients found no association between current TNFi use and obesity or overweight status [13]. Our study found no statistically significant difference in obesity categories based on the medications used by patients.

Our study has several limitations. Firstly, it is a retrospective analysis evaluating JIA patients over approximately a 12-year period, and data losses may have influenced the results, and also the ACR guidelines evolved over the study period, variations in management strategies may have occurred, potentially influencing the results. Secondly, height and weight measurements were conducted by trained healthcare personnel as part of routine care in the same setting. However, a strict protocol for detailed anthropometric assessments, including skinfold thickness, arm and waist circumference, was not followed, nor was a body composition analyzer used. This limitation prevented the evaluation of changes related to body composition. Nevertheless, the strength lies in its broad analysis covering 12 years of experience with 173 JIA patients. We believe that our study, conducted with a substantial number of patients and a long follow-up duration, will provide significant contributions to the literature.

In conclusion, our study comprehensively evaluated the effects of weight, height, and BMI parameters on disease activity and involved joints in JIA patients. Our data indicate that baseline obesity was associated with worse disease activity during follow-up and was identified as a risk factor for poorer disease control, particularly evident at the 6-month follow-up visit. Moreover, in specific subgroups, particularly in oligoarticular JIA patients, we found that higher BMI at diagnosis was associated with higher JADAS-27. The lower BMI values observed in patients with hip involvement may be linked to a more severe disease course. Indeed, improvement in linear growth was also observed in our cohort with effective treatment. Since JIA is a chronic disease and requires long-term treatment, BMI and even body composition monitoring is important and patients with low or high BMI should be given the necessary support.

## Supplementary Information

Below is the link to the electronic supplementary material.

Supplementary file1 (DOCX 23 KB)

## Data Availability

No datasets were generated or analysed during the current study.
