# Supplementary material for: The impact of BMI on disease activity and growth outcomes in juvenile idiopathic arthritis
Source: Eur J Pediatr. 2025 Mar 19;184(4):259. doi: 10.1007/s00431-025-06084-x (PMC11923038; doi:10.1007/s00431-025-06084-x)
Supplement: Supplementary file 1 — Supplementary file1 (DOCX 23 KB) [file 431_2025_6084_MOESM1_ESM.docx]

**Supplementary Table-1: Comparison of weight, height, and BMI SDS according to joint involvement**

|  |  | Weight SDS  (diagnosis) | Height SDS  (diagnosis) | BMI SDS (diagnosis) |  |
| --- | --- | --- | --- | --- | --- |
|  |  |  |  |  |  |
| Hip involvement | No (n=127) | -0,33 ± 1.63 | -0,25 ± 1.15 | -0.19 ± 1.18 |  |
|  | Yes (n=46) | -0,10 ± 1.54 | -0.56 ± 1.40 | -0.70 ± 1.93 |  |
|  | p-value* | 0,47 | 0,2 | **<0.0001** |  |
| Ankle involvement | No (n=100) | -0,37 ± 1.78 | -0.18 ± 1.18 | -0.22 ± 1.47 |  |
|  | Yes (n=73) | -0.14 ±1.32 | -0.23 ± 1.28 | -0.69 ± 1.35 |  |
|  | p-value* | 0.459 | 0.319 | 0.889 |  |
| Knee involvement | No (n=52) | 0.02 ± 1.28 | -0.14 ± 1.07 | 0.18 ± 1.57 |  |
|  | Yes (n=121) | -0.40 ± 1.71 | -0.23 ± 1.28 | -0.30 ± 1.33 |  |
|  | p-value* | 0.710 | 0.080 | 0.921 |  |
|  |  | **Weight SDS**  **(1^st^ year)** | **Height SDS (1^st^ year)** | **BMI SDS (1^st^ year)** |  |
| Hip involvement | No (n=103) | -1.75 ± 1.24 | -0.27 ± 1.22 | -0.05 ± 1.29 |  |
|  | Yes (n=40) | -0.21 ± 1.61 | -0.17 ± 1.24 | -0.25 ± 1.63 |  |
|  | p-value* | 0.084 | 0.882 | **0.049** |  |
| Ankle involvement | No (n=83) | -0.21 ± 1.30 | -0.19 ± 1.21 | -0.22 ± 1.45 |  |
|  | Yes (n=60) | -0.14 ± 1.41 | -0.32 ± 1.23 | 0.04 ± 1.30 |  |
|  | p-value* | 0.652 | 0.675 | 0.375 |  |
| Knee involvement | No (n=41) | -0.00 ± 1.30 | -0.16 ± 1.00 | 0.03 ± 1.37 |  |
|  | Yes (n=102) | -0.26 ± 1.37 | -0.28 ± 1.30 | -0.17 ± 1.41 |  |
|  | p-value* | 0.831 | 0.059 | 0.998 |  |
|  |  | **Weight SDS**  **(2^nd^ year)** | **Height SDS**  **(2^nd^ year)** | **BMI SDS (2^nd^ year)** |  |
| Hip involvement | No (n=90) | -0.10 ± 1.31 | -0.17 ± 1.20 | -0.06 ± 1.46 |  |
|  | Yes (n=30) | -0.03 ± 1.70 | 0.13 ± 1.39 | -0.13 ± 1.65 |  |
|  | p-value* | 0.260 | 0.927 | 0.531 |  |
| Ankle involvement | No (n=62) | -0.13 ± 1.34 | -0.06 ± 1.25 | -0.18 ± 1.52 |  |
|  | Yes (n=58) | -0.003 ± 1.34 | -0.13 ± 1.27 | 0.02 ± 1.49 |  |
|  | p-value* | 0.386 | 0.860 | 0.733 |  |
| Knee involvement | No (n=31) | 0.02 ± 1.22 | -0.17 ± 0.98 | 0.10 ± 1.29 |  |
|  | Yes (n=89) | -0.10 ± 1.48 | -0.07 ± 1.34 | -0.14 ± 1.57 |  |
|  | p-value* | 0.282 | **0.041** | 0.096 |  |
|  |  | **Weight SDS**  **(last visit)** | **Height SDS**  **(last visit)** | **BMI SDS (last visit)** |  |
| Hip involvement | No (n=123) | -0.27 ± 1.37 | -0.06 ± 1.35 | -0.28 ± 1.33 |  |
|  | Yes (n=45) | -0.02 ± 1.56 | 0.13 ± 1.07 | -0.22 ± 1.57 |  |
|  | p-value* | 0.446 | 0.182 | 0.152 |  |
| Ankle involvement | No (n=97) | -0.21 ± 1.21 | 0.14 ± 1.37 | -0.34 ± 1.24 |  |
|  | Yes (n=71) | 0.14 ± 1.37 | -0.22 ± 1.12 | -0.16 ± 1.59 |  |
|  | p-value* | **0.015** | 0.255 | 0.129 |  |
| Knee involvement | No (n=50) | -0.05 ± 1.31 | -0.02 ± 0.94 | -0.10 ± 1.4 |  |
|  | Yes (n=118) | -0.27 ± 1.47 | -0.002 ± 1.40 | -0.33 ± 1.39 |  |
|  | p-value* | 0.448 | 0.100 | 0.767 |  |

**Supplementary Table-2: Comparison of BMI change over years by medication use and affected joint**

| Comparison of BMI change over years by medication use | | | | | |
| --- | --- | --- | --- | --- | --- |
|  |  | Decreased BMI | Same BMI | Increased BMI | p-value* |
| NSAIDs | No | 0 | 6 | 0 | 0.209 |
|  | Yes | 31 | 109 | 27 |  |
| cDMARDs | No | 3 | 22 | 5 | 0.460 |
|  | Yes | 28 | 93 | 11 |  |
| anti-TNF | No | 20 | 69 | 19 | 0.585 |
|  | Yes | 11 | 46 | 8 |  |
| anti IL-6 | No | 29 | 112 | 26 | 0.582 |
|  | Yes | 2 | 3 | 1 |  |
| Corticosteroids | No | 25 | 80 | 19 | 0.472 |
|  | Yes | 6 | 35 | 8 |  |
| Comparison of BMI change over years by affected joint | | | | | |
| Hip involvement | No | 22 | 85 | 20 | 0.944 |
|  | Yes | 9 | 30 | 7 |  |
| Ankle involvement | No | 15 | 69 | 16 | 0.502 |
|  | Yes | 16 | 46 | 11 |  |
| Knee involvement | No | 7 | 38 | 7 | 0.465 |
|  | Yes | 24 | 77 | 20 |  |
| CRP | Normal (<5mg/L) | 12 | 41 | 8 | 0.736 |
|  | High  (≥5 mg/L) | 18 | 66 | 18 |  |
| ESR | Normal (<20mm/h) | 12 | 49 | 5 | 0.086 |
|  | High (≥20mm/h) | 18 | 59 | 19 |  |

**NSAIDs: Non-steroidal anti-inflammatory drugs, cDMARDs: conventional disease modifying anti-rheumatic drugs, anti-TNF: anti-tumor necrosis factor, anti-IL-6: anti interleukin-6*
